# Torsion of right middle lobe after a right upper lobectomy

**DOI:** 10.1186/1749-8090-4-16

**Published:** 2009-04-16

**Authors:** Chih-Hao Chen, Tzu-Ti Hung, Tung-Ying Chen, Hung-Chang Liu

**Affiliations:** 1 Department of Thoracic Surgery, Mackay Memorial Hospital, Taipei City, Taiwan; 2 Department of Pathology, Mackay Memorial Hospital, Taipei City, Taiwan

## Abstract

Lobar torsion after lung resection is a quite rare complication. A 50-year-old woman presented typical features on chest radiographs and CT(computed tomography) scan of lobar torsion after a right upper lobectomy. After emergency lobectomy of right middle lobe, the patient recovered well and discharged 10 days after the second operation.

## Background

Resection of right upper lobe is a common procedure in patients with lung cancer. Infrequently, the fissure between the middle lobe and lower lobe is well developed so that torsion of the middle lobe might occur during or after an operation, especially when pneumopexy is not performed.

## Case presentation

**Figure 1:**
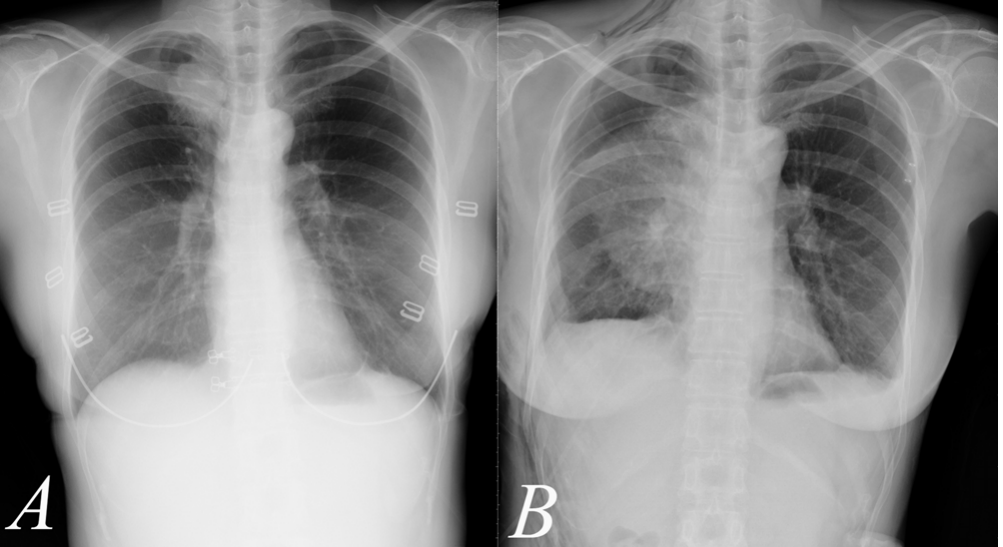
**A. mass measuring around 3.5 cm in greatest diameterin right upper lung field**. B. Follow-up chest radiograph showed a wedge-shaped opacity of large area in the middle lung field.

**Figure 2:**
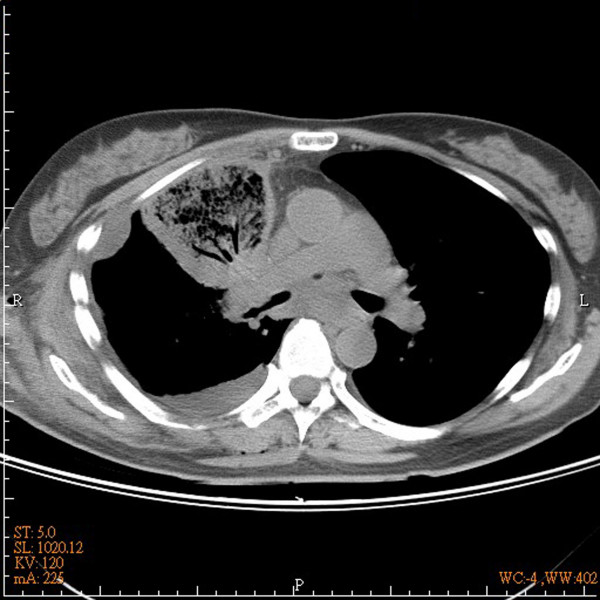
**CT scan showed collapse and hemorrhagic consolidation in right middle lobe**.

A 50-year-old woman presented 2-month cough and mild symptoms of hemoptysis. On routine chest radiograph of regular health examination, the patient was found to have a mass measuring around 3.5 cm in greatest diameterin right upper lung field (Figure 1A). Subsequent bronchoscopic brushing cytology confirmed the mass to be malignant. 7 days after admission, the patient underwent lobectomy and radical lymph node dissection through a standard right posterolateral thoracotomy. The chest radiograph on postoperative day 1 and 2 showed adequate lung expansion with no obvious abnormality. 5 days after operation, the patient presented fever and mild dyspnea. Follow-up chest radiograph showed a wedge-shaped opacity of large area in the middle lung field (Figure 1B). Bedside bronchoscopy showed tight orifice of right middle lobe. CT scan showed collapse and hemorrhagic consolidation in right middle lobe (Figure 2). Under the impression of torsion, the patient underwent explorative thoracotomy. Under direct visualization, the right middle lobe was found to have a 720-degree counterclockwise torsion along the pedicle axis. An emergency lobectomy was performed. The patient was discharged 10 days after operation.

Lobar torsion can occurred in traumatic, spontaneous and postoperative conditions. Its occurrence is quite rare [1]. Prompt diagnosis of the torsion is quite difficult and must be differentiated from sputum impaction and lobar pneumonia. Because of the other 2 conditions may response well to chest physical therapy and effective antibiotic therapy respectively. The physical findings, including fever, tachycardia, dyspnea and decreased breath sounds, are not specific to make a diagnosis. The key steps are radiographic and bronchoscopic. Typical radiographic findings include homogenous consolidation in plain film and absence of contrast enhancement in the affected lobe on CT scan [2]. A careful bronchoscopic examination may reveal abnormally tight and obstructed orifice of the affected lobe. Right middle lobe is by far the most common afftected lobe. Pneumopexy is a key procedure to prevent such complication.

## Conclusion

Pneumopexy with the use of sutures to anchor the middle lobe to the lower lobe after a right upper lobectomy must be practiced in all cases to reduce the risk of lobar torsion, especially if the fissure is well developed.

## Consent

Written informed consent was obtained from the patient for publication of this case report and any accompanying images. A copy of the written consent is available for review by the Editor-in-Chief of this journal.

## Competing interests

The authors declare that they have no competing interests.

## Authors' contributions

CHC: the main author to write the article. TTH: collection of data and help us to revise the manuscript. TYC: help us for clinical pathological interpretation. HCL: final approval of the manuscript.
